# A Synchrotron Radiation Photoemission Study of SiGe(001)-2×1 Grown on Ge and Si Substrates: The Surface Electronic Structure for Various Ge Concentrations

**DOI:** 10.3390/nano12081309

**Published:** 2022-04-11

**Authors:** Yi-Ting Cheng, Hsien-Wen Wan, Jueinai Kwo, Minghwei Hong, Tun-Wen Pi

**Affiliations:** 1 Graduate Institute of Applied Physics and Department of Physics, National Taiwan University, Taipei 10617, Taiwan; d06245005@ntu.edu.tw (Y.-T.C.); f04222061@ntu.edu.tw (H.-W.W.); 2 Department of Physics, National Tsing Hua University, Hsinchu 30013, Taiwan; 3 National Synchrotron Radiation Research Center, Hsinchu 30076, Taiwan

**Keywords:** SiGe(001)-2×1, synchrotron radiation photoemission, Si(001)-2×1, Ge(001)-2×1, low-energy electron diffraction

## Abstract

Beyond the macroscopic perspective, this study microscopically investigates Si_1−x_Ge_x_(001)-2×1 samples that were grown on the *epi* Ge(001) and *epi* Si(001) substrates via molecular-beam epitaxy, using the high-resolution synchrotron radiation photoelectron spectroscopy (SRPES) as a probe. The low-energy electron diffraction equipped in the SRPES chamber showed 2×1 double-domain reconstruction. Analyses of the Ge 3d core-level spectra acquired using different photon energies and emission angles consistently reveal the ordered spots to be in a Ge–Ge tilted configuration, which is similar to that in *epi* Ge(001)-2×1. It was further found that the subsurface layer was actually dominated by Ge, which supported the buckled configuration. The Si atoms were first found in the third surface layer. These Si atoms were further divided into two parts, one underneath the Ge–Ge dimer and one between the dimer row. The distinct energy positions of the Si 2p core-level spectrum were caused by stresses, not by charge alternations.

## 1. Introduction

Metal–oxide–semiconductors (MOSs) inevitably encounter issues related to the MO and OS interfaces. Oxide bonding at the OS interface can be optimized to achieve desirable interfacial electrical properties when the atomic and electronic structures of the semiconductors are understood. Si has been the channel material of complementary MOS (CMOS) devices for over fifty years because a perfected SiO_2_/Si interface exhibits very low interfacial trap densities. With higher carrier mobility than Si, SiGe (including Ge) is an alternative channel material in the CMOS technology [1,2,3]. Intensive research efforts on oxide/Ge and /SiGe heterostructures have led to low interfacial trap densities of 10^10^–10^11^ eV^−1^ cm^−2^ [4,5,6,7] but have not improved the device reliability [8,9,10]. Systematic studies on the electronic structure of a wide range of the Ge contents in the SiGe films grown on the Ge(001) and Si(001) substrates are still lacking. In comparison, Si and Ge have been the focus of many studies over the decades [11,12,13,14,15,16,17,18]. Among the assorted orientations of Si and Ge, the (001) surface has been studied the most. However, much work remains before we can confirm how the surface can be reconstructed and how the electronic structure should be properly interpreted. Specifically, the bulk terminated surface couples the two nearby atoms to become a 2×1 unit cell at room temperature; these atoms are further buckled, leading to one atom moving upward and the other atom moving downward. The charge is redistributed between the buckled dimer, with the up-dimer atom enriched in charge and the down-dimer atom deficient in charge. Core-level photoemission is able to differentiate the dimerized atoms by revealing two distinct peaks with resolvable energy separation in the Si 2p and Ge 3d core-level spectra. Furthermore, reconstruction on the topmost surface unavoidably affects the first subsurface layer, introducing a corresponding component into the acquired core-level spectra, making the acquired spectra even more complicated. If the second subsurface layer is involved, the analysis must extract the contributions of each surface layer from the photoemission data. This procedure is necessary for the Si 2p core-level spectrum of Si(001)-2×1.

All resolvable lines refer to the lines associated with the solid bulk in core-level photoemissions. Intuitively, a charge-enriched atom should appear with an energy lower than the bulk line; the sign of the shift is commonly called a negative shift. In contrast, a charge-deficient atom is expected to exhibit higher binding energy than the bulk with a positively shifted value. Researchers who utilize synchrotron radiation as the excitation source for high-resolution core-level photoemissions change the impinging energies and emission angles at a given photon energy to ensure a complete dataset. The goal of this method is to determine where the dimerized surface components might possibly be located in the spectra. These components show great intensity enhancements, with significant surface sensitivity and notable reductions in strength in most bulk-sensitive scans. Initial estimates of the locations of these components in the spectra are essential in a fitting analysis. However, for an atomically clean Si(001)-2×1 surface, the high-energy region of the Si 2p core-level spectrum with charge-deficient down-dimer atoms failed to increase akin to the low-energy region for the signal of charge-enriched up-dimer atoms [12,14,17,19]. Indeed, the region between the up-dimer line and bulk line agrees with these expectations. The follow-up data analysis, along with theoretical calculations based on the final-state screening effect, concurred with the data-based observations.

The Si 2p core-level spectrum of Si(001)-2×1 is yet to be fully determined because of the contributions from the first and second subsurface layers. To solve this problem, we proposed a fitting algorithm based on the physical phenomenon in photoemissions called the inelastic mean-fee path (IMFP). An IMFP was evaluated using a formulation that takes into account the actual layer-wise occupancies in the reconstructed surface [14,19]. Here, the top *N* surface-like layers are treated individually, and the rest are considered as bulk. The signal from each layer is governed by *x^N^*^−1^(1 − *x*), where *x* = exp(−2*d*/λ) for normal emissions, λ is the inelastic mean-free path (IMFP), and *d* is the layer spacing.

The IMFP method can be used to judge the areal intensity of a fit if the intensity does not properly meet the expected values. In this study, the line shape of the Si 2p core-level spectrum was composed of two components that originated from the dimers: one from the first subsurface layer, and two from the second subsurface layer. As mentioned above, the two surface components were analyzed considering a charge imbalance. The application of this process to the second subsurface layer was met with great difficulty. According to a known reconstruction, the atomic model of Si(001)-2×1 indicates that the second subsurface layers are different when they lie directly below a row of buckled dimers and when they lie between the dimer rows. These types of layers endure different stresses. Atoms underneath the dimers experience compressive stress, whereas those between the dimers experience tensile stress. Consequently, two distinctive lines, with one shifted negatively and the other positively, appeared in the resolved spectra [12,14,17,20], as commonly reported in the literature.

The line shifts in the Si 2p core-level spectra have two origins: a charge effect and a stress effect. The argument for properly interpreting the Si 2p core-level spectrum of Si(001)-2×1 is valid for the Ge 3d core-level spectrum of Ge(001)-2×1, but with one exception: the stress-free second subsurface layer of Ge(001)-2×1. Ultimately, the line shape of the Ge 3d core-level spectrum is composed of two dimerized components and one subsurface component.

Si and Ge can be intermixed to become Si_1−*x*_Ge*_x_* alloys, with *x* ranging from 0 to 1. The 4.1% lattice mismatch of Si and Ge causes an intrinsic strain in SiGe alloys grown on Ge or on Si. Based on this aforementioned information, the strain should mainly be reflected on Si, rather than on Ge. Reports on specific details of surface behavior are scarce in both atomic and electronic studies. This factor is peculiar, considering that SiGe, with its high carrier mobility, represents a viable material to replace Si as the channel layer for CMOS technology in sub–3 nm nodes [1]. Indeed, we recently used high-resolution core-level photoemissions with synchrotron radiation to investigate the electronic structure of Si*_0.3_*Ge*_0.7_*(001)-2×1 grown on *epi* Ge(001) [21].

In this work, we extended this analysis to systematically study the surface electronic structure of the Si_1__−*x*_Ge*_x_*(001)-2×1 films epitaxially grown on Ge(001) and Si(001) substrates, respectively, with a wide range of Ge contents (*x* from 10% to 90%). We found that although the strain and the composition are different in all samples, the surface unit cells were basically similar in all samples based on the presence of the surface top two atomic layers being Ge atoms: the topmost surface layer is buckled Ge–Ge dimers with the charge transfer from the down-atom dimer to the up-atom dimer, and the third layer is predominately occupied by Si, followed by the fourth layer of SiGe. Note that differences exist between these cells in charge distribution, thereby producing different tilted angles. The high-κ/Si_1−*x*_Ge*_x_* interface has become one of the most critical issues with the continued scaling of CMOS technology. The present study revealed that the heterointerface of high-κ on Ge and Si_1−*x*_Ge*_x_* is practically the same, since the topmost surface of both Si_1−*x*_Ge_x_ and Ge is terminated with the Ge-Ge dimers irrespective of the Ge contents.

## 2. Experimental Section

### 2.1. Sample Preparations, Surface Structure, and Morphological Characterization

In this study, we used high-resolution core-level photoemissions to simultaneously probe the electronic structure of Si_1−*x*_Ge*_x_*(001)-2×1 *epi*-layers grown on the Ge(001) and Si(001) substrates, with *x* ranging from 10% to 90%. SiGe(001)-strained epi-layers were grown and characterized in a multi-chamber ultra-high vacuum (UHV) system, which included molecular beam epitaxy (MBE) chamber and scanning tunneling microscopy (STM) chamber [21,22]. In this work, we studied the surface roughness of the MBE as-grown SiGe films using the in situ STM in our multi-chamber system. All the experiments of film growth and surface morphology characterization were carried out in UHV to ensure the surface cleanliness of the samples.

The preparation of SiGe films on *epi* Ge(001) substrates was as follows: 50 nm thick *epi* Ge(001) layers were first grown on Ge(001) wafers using MBE. Si_1−*x*_Ge*_x_* films were then grown on the *epi* Ge(001)-2×1 substrates at 500 °C again using MBE. The thickness of the Si_1−*x*_Ge*_x_* films with *x* = 0.7 and 0.9 grown onto the *epi* Ge(001) was 10 nm, whereas for *x* = 0.1 and 0.3, it was 2 nm thick, and for *x* = 0.5, it was 4 nm thick. The process for the preparation of the SiGe films grown on *epi* Si(001) substrates was as follows: After a chemical clean by the RCA cleaning procedure followed by an HF dip, the Si(001) wafers were immediately loaded in the UHV multi-chamber growth system. Si epitaxial layers were deposited using MBE on the Si(001) substrates at 700 °C for attaining chemically clean and atomically ordered Si(001) surfaces with a 2×1 reconstruction. Si_1−*x*_Ge*_x_* films with a thickness of 1 nm were MBE-grown on the *epi* Si(001). The Si_1−*x*_Ge*_x_* films with *x* = 0.1 and 0.5 were grown at 500 °C, whereas Si*_0.1_*Ge*_0.9_* was grown at 400–450 °C to ensure that the film remained strained. Three samples with Ge contents of 0.1, 0.5, and 0.9 were prepared for the photoemission study. The sample composition was determined using high-resolution synchrotron radiation X-ray diffraction (HR-XRD).

**Figure 1:**
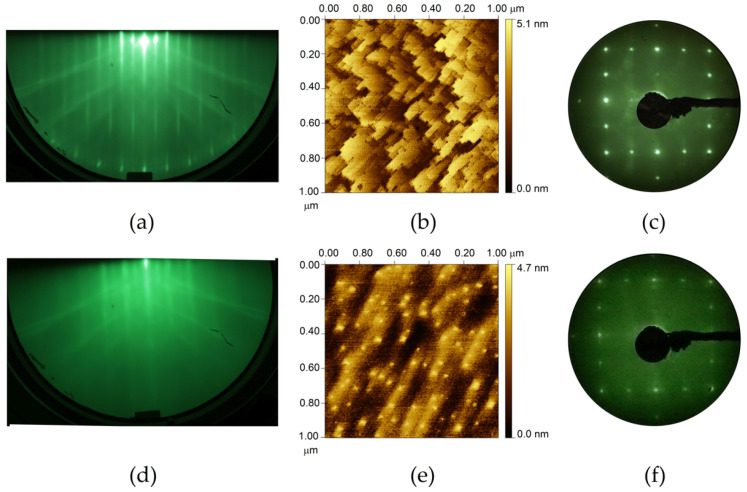
(**a**) RHEED pattern, (**b**) STM image, and (**c**) LEED pattern of MBE as-grown 4 nm–thick Si*_0.5_*Ge*_0.5_* on *epi* Ge(001)-2×1, and (**d**) RHEED pattern, (**e**) STM image, and (**f**) LEED pattern of MBE as-grown 1 nm–thick Si*_0.1_*Ge*_0.9_* on *epi* Si(001)-2–1. STM images are 1 µm × 1 µm in size. The electron beam energy of the LEED images was 40 eV.

The surface reconstruction of the SiGe/*epi* Ge(001) samples as monitored by in situ reflection high-energy electron diffraction (RHEED) shows only a 2×1 structure. Figure 1a shows the RHEED pattern of the as-grown 4 nm–thick Si*_0.5_*Ge*_0.5_* on *epi* Ge(001). In comparison, upon depositing 1 nm–thick Si*_0.1_*Ge*_0.9_* on *epi* Si(001), we observed a combination of 2×1 and 2×n reconstructions in the RHEED pattern, as shown in Figure 1d. Both SiGe samples present streaky diffraction spots and distinct Kikuchi arcs. The sharp and intense diffraction patterns indicate that these two surfaces were atomically flat and ordered. The 2×n reconstruction of the Si*_0.5_*Ge*_0.5_*/*epi* Si (001) was also observed in the low-energy electron diffraction (LEED) pattern, as shown in Figure 1f. Immediately after growth, both SiGe samples were transferred in situ via the UHV transfer module to the STM system for surface morphology characterization. Figure 1b contains a 1 µm × 1 µm STM image of the Si*_0.5_*Ge*_0.5_* on the *epi* Ge(001)-2×1. The root-mean-squared (RMS) surface roughness from the 1 µm × 1 µm image for the 4 nm–thick Si*_0.5_*Ge*_0.5_* film under tensile strain is 0.39 nm. Figure 1e shows the STM image of the highly compressively strained 1 nm–thick Si*_0.1_*Ge*_0.9_* on *epi* Si(001)-2×1, with the RMS surface roughness of 0.31 nm.

After the film growth, the samples were transferred immediately to a UHV portable chamber maintained at 2 × 10^−10^ Torr, which served to transfer the samples to Taiwan’s National Synchrotron Radiation Research Center (NSRRC) for photoemission measurements [23]. The spectral data were then collected using a 150 mm hemispherical analyzer (SPECS) in a UHV chamber with a base pressure of 2 × 10^−10^ Torr or less. The Ag energy references were mounted on a metal sample holder, and the instrument resolution was better than 60 meV. The surface reconstruction of the SiGe films was checked by LEED. Representative images of the LEED in the photoemission chamber are shown in Figure 1c,f for the Si*_0.5_*Ge*_0.5_*/*epi* Ge(001) and Si*_0.1_*Ge*_0.9_*/*epi* Si(001) substrates, respectively. The sharp spots indicate a 2×1 double-domain reconstruction. It is worth mentioning that SiGe films grown on *epi* Ge(001)-2×1 substrate exhibit 2×1 reconstruction only irrespective of the Ge contents. The Si_1−*x*_Ge*_x_*(001)/*epi* Si(001) sample with 10% Ge content shows only 2×1 reconstruction, while 2×n reconstruction along with 2×1 reconstruction was imaged for the samples with 50% and 90% Ge content. The appearance of a 2×*n* structure in the Si_1−*x*_Ge*_x_*/Si(001) was reported previously [24].

### 2.2. Data Analysis

The objective of the synchrotron radiation photoelectron spectroscopy (SRPES) experiments on semiconductor surfaces was to relate the observable features to the known properties of the reconstructed surfaces. The model function mainly consists of the polynomial background function, the Voigt function, and the gap-excitation function. The polynomial function is generally sufficient for the structureless inelastic scattering but becomes insufficient for semiconductors with narrow bandgaps. Ejection of a core-level electron could introduce a disturbance of electrons about the valence band maximum to result in a bandgap excitation. A photoemission component is commonly represented by a Voigt function line, which is a convolution of the Lorentzian and Gaussian functions. Constraints are necessary to reduce the ambiguity of a fit, such as the lifetime width, spin–orbit splitting, and spin–orbit ratio being essentially identical for all components. The three parameters are, for each spin–orbit pair, the position, height, and Gaussian width. The areas of Voigt function lines are not proportional to the product of peak height and Gaussian width; therefore, it is necessary to numerically integrate the area to set the peak amplitude.

## 3. Results and Discussion

### 3.1. Valence Band Spectra of SiGe Alloys

**Figure 2:**
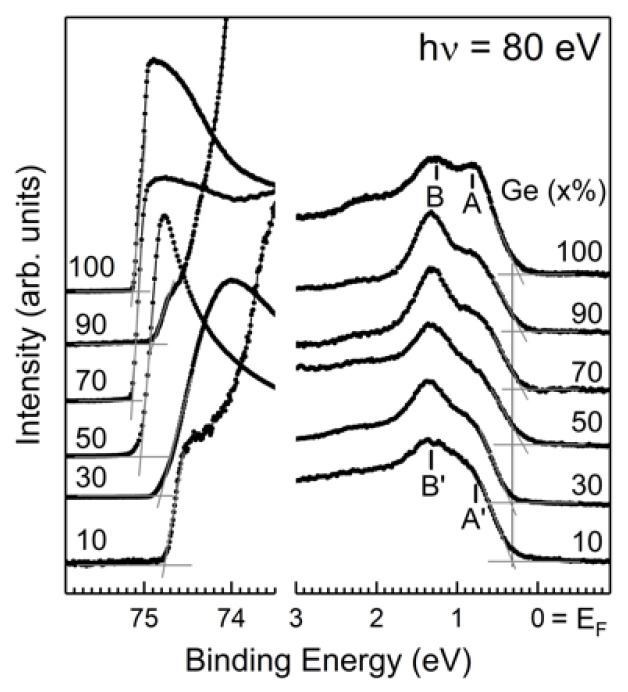
Photoemission data from the molecular beam epitaxy (MBE) as-grown Si_1−*x*_Ge*_x_*(001)-2×1 for the valence band spectra and the cutoffs. Symbols A and B stand for the Ge 4p states of Ge(001). Features A’ and B’ both contributed from Si 3p and Ge 4p states of Si_1-x_Ge_x_(001).

Figure 2 displays the valence band and cutoff region with 80 eV photon energy. The valence band maximum (VBM) could be determined by extrapolating the leading edge of the valence band spectrum with a constant structureless background; the value was 0.25 eV below the Fermi level (E_F_). The ionization potential (IP) in a semiconductor is determined by measuring the spectral width (W) at given photon energy, where W is the energy separation of the VBM and the cutoff of the photo-ejected electrons. The IP value is determined by subtracting hν from W. The ionization potentials for various *x* contents are listed in Table 1. All values were directly obtained from the acquired spectra without any assumptions. For Ge, the distinct features A and B right below VBM originated from the Ge 4p states. For Si_1−*x*_Ge*_x_* film with *x* ranging from 10% to 90%, features A’ and B’ both originated from Si 3p and Ge 4p states. The change of the Ge content in the SiGe is reflected in the relative intensities of features A’ and B’.

### 3.2. General Analysis of Core-Level Spectra

**Figure 3:**
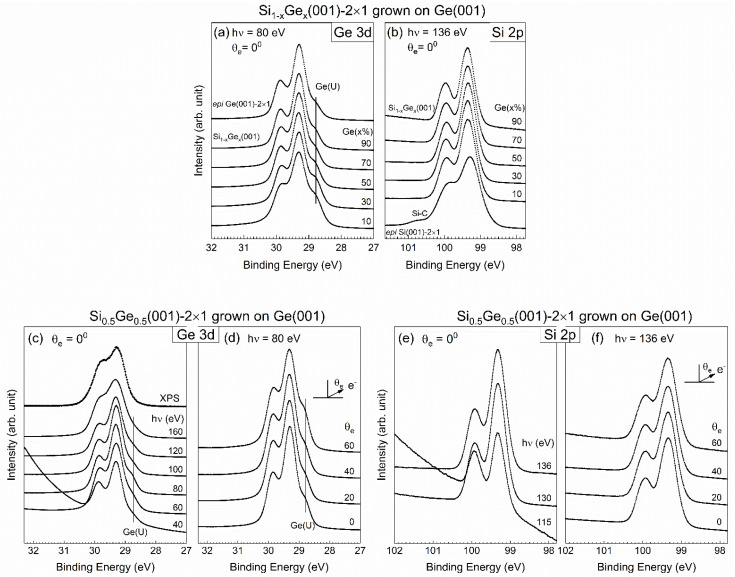
Photoemission data from the as-grown Si_1−*x*_Ge*_x_*(001)-2×1 on an *epi* Ge(001) substrate with the Ge contents ranging from 10% to 90% for the Ge 3d (**a**) and Si 2p (**b**) core-level spectra. The Ge 3d states of the representative *x* = 50% sample were acquired at (**c**) various photon energies (hν) taken under normal emissions (θ_e_ = 0°) at room temperature, as well as at (**d**) various emission angles at hν = 80 eV. Symbols Ge(U) stand for Ge up-dimer counterpart. The Si 2p states of the representative *x* = 50% sample were acquired at (**e**) various photon energies (hν) taken under normal emissions (θ_e_ = 0°) at room temperature, as well as at (**f**) various emission angles at hν = 136 eV.

Wide scans were taken of the as-grown Si_1−*x*_Ge*_x_*(001)-2×1 samples featuring various Ge contents with 136 eV photon energy (hν) and simultaneously covering both the Si 2p and Ge 3d core-level spectra under normal emissions at room temperature. These scans showed the increased strength of the Ge 3d line with an increase in *x* (data not shown). The details for the Ge 3d and Si 2p core-level spectra with a good signal-to-noise ratio are presented in Figure 3, with *x* ranging from 10% to 90% in panels Figure 3a,b, respectively. The single elements of *epi* Ge(001)-2×1 (*x* = 1) and *epi* Si grown on *epi* Ge(001)-2×1 (*x* = 0) are also included in the plots as references. The photon energies for the Ge 3d and Si 2p core-level spectra were 80 and 136 eV, respectively. We selected a sample with 50% Ge content as the representative sample upon excitation with various photon energies and emission angles. The acquired Ge 3d core-level spectra are shown in Figure 3a,c,d, and the Si 2p core-level spectra are provided in Figure 3b,e,f. First, we discuss the behaviors of the Ge 3d core-level spectra. Notably, the data were collected after the MBE-grown SiGe(001)-2×1 sample had been docked in the portable UHV module for 12 h. The absence of an oxidation state in both the Si and Ge core-level spectra suggested that the SiGe(001) surface was surprisingly stable in a vacuum, as Si(001)-2×1 surfaces are readily oxidized under similar UHV conditions. Stable surfaces were also found on the MBE-grown *epi* Ge(001)-2×1 [18,25,26] and (In)GaAs(001) surfaces [23,27,28,29,30]. The binding energies of the bulk Si 2p and Ge 3d core-levels were located at 99.31 and 29.26 eV, respectively. The energy position of the Ge 3d state was found to coincide with that in *epi* Ge(001)-2×1 [18,25,26], although the energy position of the Si 2p state appeared to be noticeably lower than the nominal value of 99.8 eV in crystalline Si(001)-2×1 [31].

### 3.3. Specifics for Ge 3d Core-Level Spectra

As shown in Figure 3a, the Ge 3d core-level spectra in Si_1−*x*_Ge*_x_*(001)-2×1 grown on *epi* Ge(001)-2×1 remained similar in line shape irrespective of the Ge contents. All spectra show a characteristic bump in the low-energy region. In *epi* Ge(001)-2×1, this feature is known to be caused by emissions from the up-dimer atom in the reconstructed buckled dimer. The guide lines in Figure 3a,c suggest similar origins for the Si_1−*x*_Ge*_x_*(001)-2×1 samples. In Figure 3c, the line ends with 160 eV photon energy without extending to the XPS spectrum because the features of the up-atom dimer become noticeably smaller, and the technique is unable to clearly resolve this issue. The low-energy feature emerged from the surface Ge, which was further justified by the enhancement in intensity under off-normal emissions (see Figure 3d).

The surface reconstruction of Si(001)-2×1 and Ge(001)-2×1 ultimately assumed the form of buckled dimers, with one atom moving upward and the other moving downward. The charge imbalance between the dimerized atoms was reflected in the core-level spectra at different energy positions [12,14,17,19]. The small energy shifts of the components contributed by the bulk line mingled together into a broad line envelope. This made it difficult to differentiate between the initial-state effect and the final-state effect because of the screening mechanisms for the Si 2p core-level spectrum in Si(001)-2×1 and the Ge 3d core-level spectrum in Ge(001)-2×1, as both effects were present in the line spectrum. A theoretical calculation was used to validate the final-state effect as the primary screening mechanism [13]. This final-state effect involves a crystal ensemble with a created core hole and a photo-excited electron. This effect is hypothesized to represent the unoccupied dangling-bond state contributed by down-dimer atoms pulled down because of the influence of a core hole. The down-shifted dangling-bond state then becomes populated by electrons at the Fermi level, thereby presenting smaller binding energy than the initial-state effect. Experimentally, a core-level line associated with the down-dimer atoms was buried a few tenths of electronvolt away from the bulk, underneath it. This made a visual inspection of core-level line an impossible task, instead of requiring a fit to extract the line.

**Figure 4:**
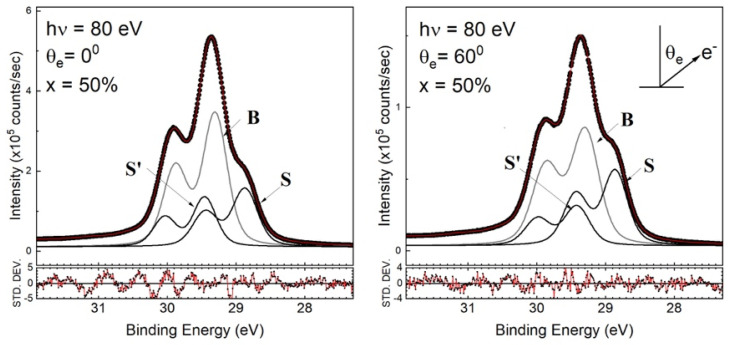
Fit to the Ge 3d core-level spectra with the 50% Ge content of Si_1−*x*_Ge*_x_*(001)-2×1. θ_e_ is the emission angle with respect to the surface normal. Symbols S and S’ stand for Ge up-dimer and Ge down-dimer counterparts. Symbol B stands for the Ge bulk component.

Before the final-state effect validated the interpretation of the Ge 3d (Si 2p) core-level spectrum, researchers often studied the final-state effect based on the initial-state effect. This practice was naturally applied to the Ge 3d and Si 2p core-level spectra acquired for SiGe(001), as reported in Ref. [32]. As a result, this previous study claimed that the topmost SiGe(001) surface was terminated with buckled Ge–Ge dimers and that the subsurface layer was occupied by Si. The initial-state effect was initiated to assign the features with lower binding energy than that of the bulk line. This lower energy corresponds to the up-dimer atoms (S), whereas the down-dimer counterparts (S’) have greater binding energy. The researchers in Ref. [32] used a deconvolution method to obtain a conclusion without further employing fitting to explicitly present the dimerized components. We tentatively made a similar assumption to process our Ge 3d core-level spectra data, which featured a higher-energy resolution than the data in Ref. [20]. The fitted results for *x* = 50% are shown in Figure 4. The Ge 3d line is represented by three components associated with bulk Ge(B), S, and S’ components. Upon fixing the shifted values of components S and S’, we found that the S component increased in intensity, but the S’ component instead dropped in strength under off-normal emissions. However, for components S and S’ in both angled spectra, the fitted results gave an unrealistic areal intensity ratio far from the expected value, which is one. This suggested the need to examine the validity of interpreting the Ge 3d core-level spectra with the initial-state effect. Notably, oxygen-induced features generally appear in high-energy positions. If the Ge 3d core-level spectrum is not properly understood, confusion might arise, thereby making it difficult to truly understand atom-to-atom interactions at the interface.

**Figure 5:**
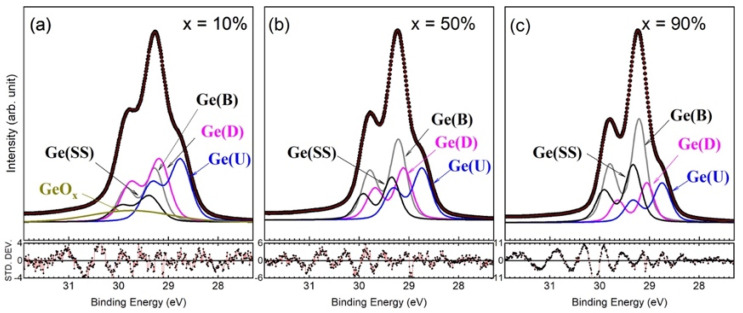
Fit to the Ge 3d core-level spectra for *x* = 10% (**a**), 50% (**b**), and 90% (**c**).

As shown in Figure 3a, the line shape of the Ge 3d core-level spectrum is practically identical to that of *epi* Ge(001)-2×1 [18,25,26]. Hence, we employed the model function for the Ge 3d core-level spectrum of *epi* Ge(001)-2×1 to analyze the representative Ge 3d core-level spectrum in Figure 3a, the results of which are shown in Figure 5. As a result, the Lorentzian width was 0.157 ± 0.005 eV. The bulk Gaussian width reached as high as 0.330 eV for *x* = 10% but gradually decreased in magnitude with increasing *x* concentrations, ending at 0.260 eV for *x* = 90%. As shown in Figure 5 and Table 2, the bulk Ge(B) component behaved as expected and presented less content with a low *x* value but gradually increased the concentration by increasing *x*. Nevertheless, the areal intensity of the component Ge(B) in *x* = 90% was smaller than that in *epi* Ge(001)-2×1.

**Table 2:** Fitted parameters of the Ge 3d and Si 2p core-level spectra in Figure 5 and Figure 6. SCLS stands for the surface core-level shift.

	*x* = 10%	*x* = 30%	*x* = 50%	*x* = 70% [21]	*x* = 90%	*epi* Ge(001)
**Ge 3d**						
SCLS of Ge(SS)	+0.106	+0.100	+0.132	+0.110	+0.118	+0.101
SCLS of Ge(U)	−0.519	−0.523	−0.476	−0.464	−0.463	−0.468
SCLS Ge(D)	−0.094	−0.130	−0.105	−0.142	−0.157	−0.090
Δ(Ge(U)-Ge(D))	0.425	0.393	0.371	0.322	0.306	0.378
% Area (Ge(B))	21%	28%	34%	40%	41%	46%
**Si 2p**						
Δ(Si(S)’-Si(B))	0.170	0.168	0.144	0.187	0.178	
Δ(Si(S)”-Si(B))	0.128	0.123	0.135	0.143	0.197	
% Area (Si(B))	48%	41%	55%	61%	61%	

The fitted values of the surface core-level shifts (SCLSs) of the Ge(U), Ge(D), and Ge(SS) components are presented in Table 2. The fit was obtained using the results of parameter correlations less than 0.98 and a χ^2^ value near to 1. The SCLSs of the dimerized components here depend on the Ge concentration. When the concentration becomes denser, the shifted value decreases in magnitude for the up-dimer component, Ge(U), but increases for the down-dimer component, Ge(D). Interestingly, the energy separation, Δ(Ge(U)-Ge(D)), becomes smaller with an increase in Ge concentration. This behavior suggests that the tilted angle of the buckled surface dimer becomes smaller with an increase in Ge concentration. In Table 2, the dimer configuration of Si_1−*x*_Ge*_x_*(001)-2×1 that best conforms with that of *epi* Ge(001)-2×1 is located at *x* = 50%.

### 3.4. Specifics for Si 2p Core-Level Spectra

In Figure 3, the as-deposited Si 2p core-level spectra modeled with different x, hν, and θ_e_ values visually present a simple line shape for the spin–orbit splitting state. The spectra show dissimilar line shapes to those of *c*-Si(001)-2×1. Notably, the lack of an up-dimer atom intrudes sharply into the lowest binding energy of the spectrum [12,14,19]. This result fulfills the assumption of total Ge segregation onto the SiGe surface with the total absence of Si atoms [33,34,35]. Thus, the Si atoms in SiGe simply behave as the bulk. This assumption can be easily tested based on a preliminary fit with only one component, with the result shown in Figure 7, where the standard deviation curve plotted in the lower panel shows severe structural fluctuation, and χ^2^ runs as high as 77. Moreover, the spin–orbit splitting ratio falls short of the expected 0.5. These unsatisfactory results suggest that one electronic environment associated with bulk Si is insufficient to show the electronic structure of SiGe(001).

**Figure 6:**
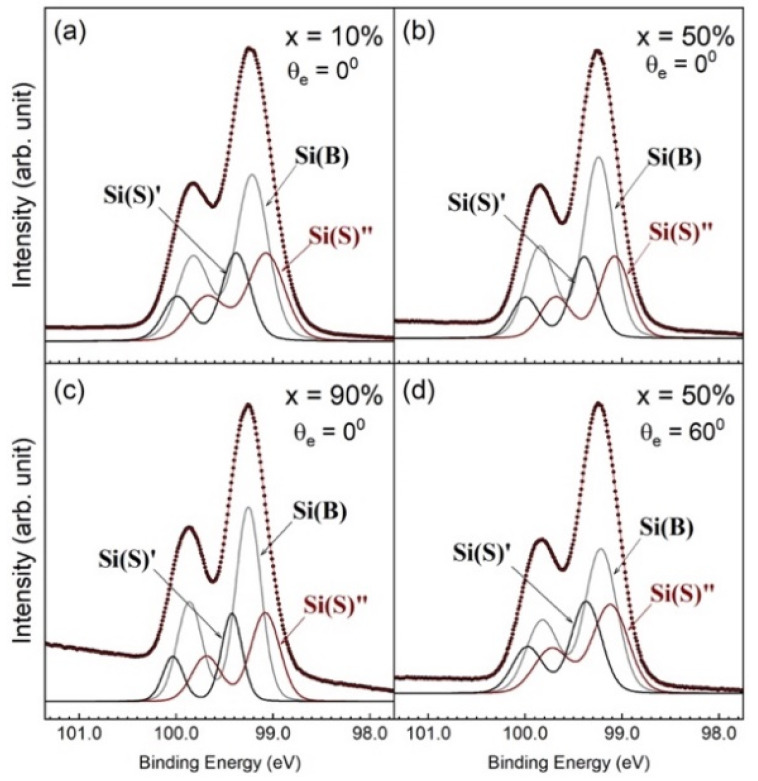
Fit to the Si 2p core-level spectra for *x* = 10% (**a**), 50% (**b**), and 90% (**c**) under normal emissions (θ_e_ = 0°). The 60° off-normal emissions for x = 50% are plotted in (**d**).

The spectral line requires three components to achieve a consistent fit for all the acquired Si 2p core-level spectra. We employed the model function to analyze the Si 2p core-level spectra in Figure 3b,f, and the results are plotted in Figure 6. In the fit, the spin–orbit splitting and spin–orbit ratio are 0.608 ± 0.001 and 0.506 ± 0.003, respectively. The binding energies of the Si(S)’, Si(B), and Si(S)” components presented mean values of 99.42, 99.27 and 99.12 eV, respectively. The binding energies are issued to emphasize the independence of components with stress on their own (see below).

**Figure 7:**
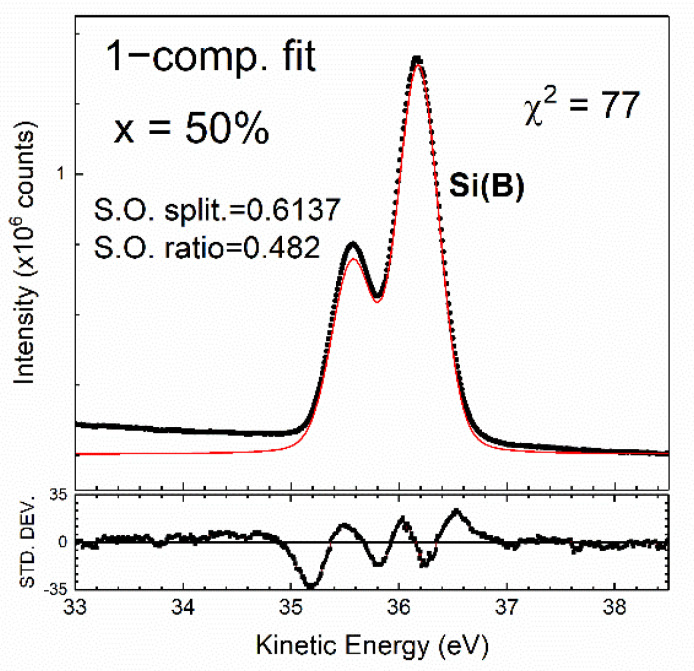
Fit to the clean Si 2p core-level spectrum based on the assumption of one component. The fitted results of χ^2^, spin–orbit splitting and the spin–orbit ratio are marked. The bottom panel presents a plot of the standard deviation of the fitted line and the spectral line. The Si(B) component corresponds to the Si atoms in the bulk of the SiGe film.

The well-established Si 2p (Ge 3d) core-level spectrum of Si(001)-2×1 (Ge(001)-2×1) reveals the emission from the top surface layers [12,14,17,19,20]. The dimers in the topmost surface layer are manifested by a charge transfer from the down-dimer atoms to the up-dimer atoms, thereby resulting in a buckled configuration. The component associated with the charge-enriched up-dimer atom presents a lower binding energy than that in the bulk as expected. However, the charge-deficient down-dimer counterpart appears in a lower binding energy than the bulk as well. This physical manifestation holds true for both Si(001)-2×1 and Ge(001)-2×1 surfaces. The anomalous behavior has been accepted as a physical phenomenon caused by the final-state effect [14], meaning that the unoccupied dangling-bond state contributed to by the down-dimer atoms is pulled down upon by the presence of a core hole created by photoemissions. The down-shifted dangling-bond state then becomes populated by electrons at the Fermi level, thereby giving rise to effective screening. The Ge(U) and Ge(D) components in Figure 5 behave similarly to the surface dimers of Ge(001)-2×1.

In Si(001)-2×1, emission from the third surface layer was also resolved in the Si 2p core-level spectrum [12,14,17]. Interestingly, they appear at different energy positions, where one exhibited a negative SCLS and the other exhibited a positive SCLS. It is not physical to attribute the opposite signs of shifts in the third surface layer as due to the charge-transfer effect. Physically, the atoms underneath the dimers in the first surface layer experience compressive stress and atoms located between the dimers experience tensile stress. Therefore, the stress effect causes the Si atoms in the third surface layer to appear in different binding energies.

Now, it is understood that the Si atoms in Si(001)-2×1 could endure either the final-state effect or the stress effect. Both should serve as references to interpret the resolved Si 2p components in Figure 6. The screening effect is not feasible here, because on the one hand, both the Si(S)’ and Si(S)” atoms would be assumed to reside on the SiGe(001) surface, and on the other hand, the initial-state effect should arise to interpret the energy positions of the Si(S)’ and Si(S)” components. Both statements clearly violate the established facts. We then suggest that components Si(S)’, Si(B), and Si(S)” with distinct energy positions are caused by stress. Both Si(S)’ and Si(S)” atoms lie right below the Ge(SS) layer, and the Si(B) atoms are found in the SiGe bulk.

### 3.5. SiGe(001)-2×1 Grown on a Si Substrate

**Figure 8:**
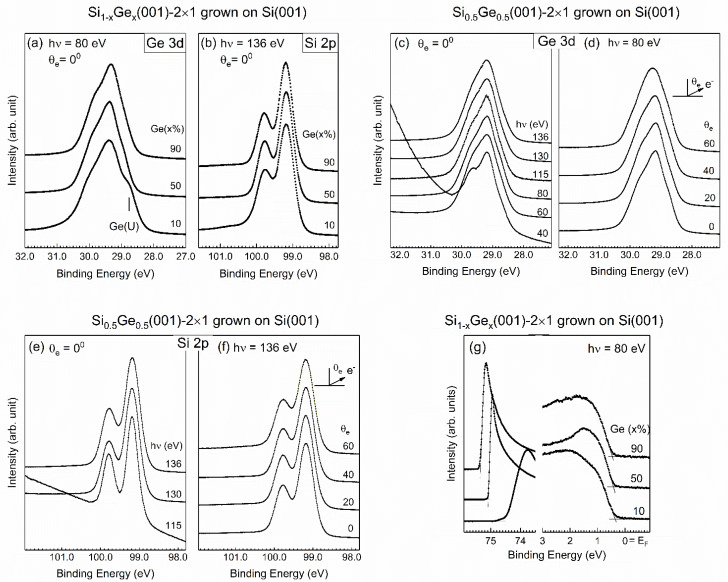
Photoemission data from the as-grown Si_1−*x*_Ge*_x_*(001)-2×1 on an Si(001) substrate with Ge contents of 10%, 50%, and 90% for the Ge 3d (**a**) and Si 2p (**b**) core-level spectra. The Ge 3d states of the representative x = 50% sample were acquired at (**c**) various photon energies (hν) taken under normal emissions (θ_e_ = 0°) at room temperature, as well as at (**d**) various emission angles at hν = 80 eV. The Si 2p states of the representative x = 50% sample were acquired at (**e**) various photon energies (hν) taken under normal emissions (θ_e_ = 0°) at room temperature, as well as at (**f**) various emission angles at hν = 136 eV. Panel (**g**) shows the valence band spectra and the cutoffs.

Next, a similar study was executed on Si_1−*x*_Ge*_x_*(001) that was grown on the Si(001) substrate. The acquired data are shown in Figure 8 for the representative Ge contents of 10%, 50%, and 90%. The IP values illustrated in Figure 8g are listed in Table 1.

**Figure 9:**
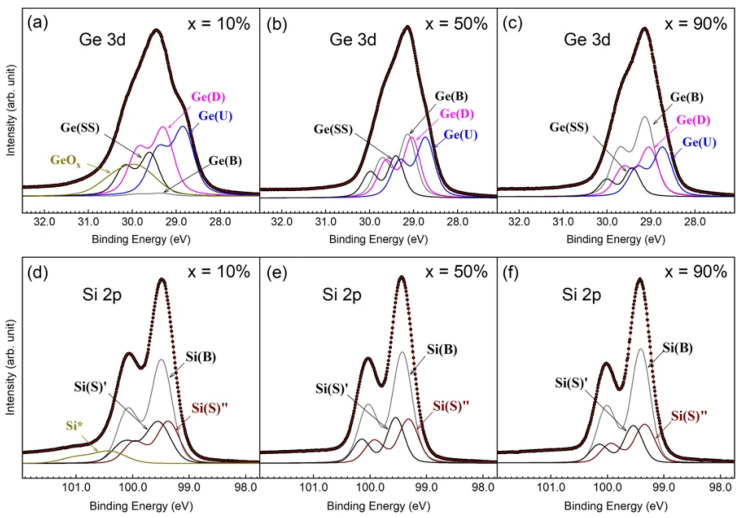
Fitting for the Ge 3d (**a**–**c**) and Si 2p (**d**–**f**) core-level spectra.

The most significant finding in the core-level spectra of Figure 8 is the line shape of the Ge 3d state, which is visually dissimilar from that shown in Figure 3. Nevertheless, the line shape of the Si 2p core-level spectra is the same as that shown in Figure 3, which suggests that the silicon atoms in Si_1−*x*_Ge*_x_*(001) supported on Si(001) were located in the third layer and below. This result suggests that the top two surface layers were still dominated by Ge. We then tentatively employed a similar model function to analyze the Ge 3d core-level spectra provided in Figure 8, and the results are presented in Figure 9a–c. Surprisingly, the fit was good when using the fewest fitted parameters, meaning that the topmost surface Ge layer was governed by buckled Ge–Ge dimers with the second Ge layer. The resulting parameters are tabulated in Table 3. For *x* = 10%, a broad line with a shift of 0.466 eV needs to be added to represent the high energy shoulder for a good fit, which is attributed to oxidized Ge. Comparing the spectral weights of the bulk components in Table 2 and Table 3, the higher percentage in the former comes mainly from the Ge(001) substrate. This phenomenon functions under the expectation that Si is able to scavenge the Ge substrate atoms on the surface to migrate up to the SiGe surface [36]. Notably, the subsurface Ge(SS) state exhibited a much greater magnitude shift, making it unrealistic to consider this state as a consequence of losing charge. Both the initial-state and final-state theories calculated the shifted value of the atoms in the subsurface layer of Ge(001) as negative. Hence, the positive values observed for SiGe(001)-2×1 and Ge(001)-2×1 suggest another origin for this shift. We propose that the subsurface Ge atoms experienced mild stress because of the surface reconstruction of the buckled dimers.

For Si 2p, a fit with three components was sufficient to represent the line spectrum (Figure 9d–f), with the fitted results shown in Table 3. As shown in Figure 9, the sample of 10% Ge was vulnerable to the chamber residuals such as CO and CO_2_. This finding suggests that this limited amount was unable to fully cover the surface with the buckled Ge–Ge dimers. In other words, the grown SiGe alloys had a limited number of silicon atoms on their surface. A minor difference was found in the Ge and Si substrate systems, especially in the samples with less Ge. For the Si(001) substrate, the 10% sample presented insufficient Ge to fully cover the SiGe surface. The small amount of Si on the SiGe surface became vulnerable to the chamber residuals, and the acquired Ge 3d and Si 2p core-level spectra exhibited oxidized features that were absent in the other samples. Nevertheless, the Si* component could have been due to plasmon loss.

**Figure 10:**
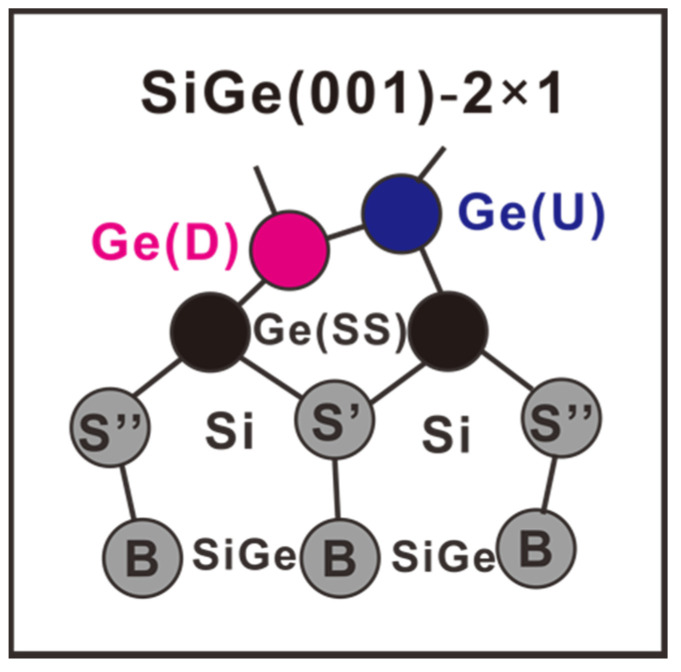
Schematic drawing of Si_1−*x*_Ge*_x_*(001)-2×1, which is good for samples grown on the Ge(001) and Si(001) substrates irrespective of the Ge contents. Symbols Ge(D), Ge(U), and Ge(SS) stand for the up-dimer Ge atoms, the down-dimer Ge atoms, and the Ge atoms in the subsurface layer, respectively. Symbols S’ and S” stand for Si atoms in the third surface layer. The symbol B stand for Si and Ge atoms in the bulk of SiGe film.

Figure 10 presents the schematic drawing of Si_1−x_Ge_x_(001)-2×1 that summarizes the results in the end, which is good for samples grown on the Ge(001) and Si(001) substrates irrespective of the Ge contents. The proposal is different from that of Ref. [37], which gave the Ge signature only on the topmost surface layer.

## 4. Conclusions

In this study, the electronic structure of Si_1−*x*_Ge*_x_*(001)-2×1 epitaxially grown on Ge(001) and Si(001) substrates, with a whole range of Ge contents, was systematically analyzed in detail using high-resolution synchrotron radiation photoemissions. The SiGe(001) alloys exhibited surface reconstruction to achieve double-domain 2×1 periodicity, as seen in the LEED image. Analysis of the Ge 3d and Si 2p core-level spectra revealed that the surface was mainly composed of Ge–Ge buckled dimers. A charge imbalance occurred between the dimerized atoms, giving rise to two distinct peaks in the Ge 3d core-level spectrum. The top Ge surface was followed immediately by a single Ge layer. The first two surface layers of the SiGe(001)-2×1 of all the studied alloys actually mimicked those of Ge(001)-2×1. Consequently, the line shapes of the Ge 3d core-level spectra were similar among Ge and all the studied SiGe alloys. The silicon atoms started to exist from the third layer. The apparent bulk position showed a distinct electronic structure compared to the positions deeper in the bulk. The Si atoms in the third layer of SiGe(001)-2×1 behaved similarly to those of Si(001)-2×1. Two components were resolved, with one related to compressive stress and the other to tensile stress. The bulk Si atoms in the *epi* Si grown on *epi* Ge also encountered stresses, which was evidenced by their smaller binding energy compared to the Si(001)-2×1. The present study revealed that the high-κ/SiGe(001) interface was the same as the high-κ/Ge(001) interface. For both interfaces, one needs to consider the responses of the Ge–Ge surface dimers to foreign elements.

## Figures and Tables

**Table 1:** Ionization potential (IP) of Si_1−*x*_Ge*_x_*(001)-2×1 grown on Ge(001) and Si(001), with *x* ranging from 10% to 90%. The IPs of Si(001)-2×1 and Ge(001)-2×1 are also listed.

	Si(001)	*x* = 10%	*x* = 30%	*x* = 50%	*x* = 70%	*x* = 90%	Ge(001)
IP (eV) (on Ge)		5.40	5.32	5.06	4.90	5.19	5.04
IP (eV) (on Si)	5.27	5.26		5.15		5.14	

**Table 3:** Fitted parameters of the Ge 3d core-level spectra in Figure 9.

	*x* = 10%	*x* = 50%	*x* = 90%
**Ge 3d**			
SCLS of Ge(SS)	+0.254	+0.269	+0.297
SCLS of Ge(U)	−0.512	−0.405	−0.398
SCLS Ge(D)	−0.056	−0.092	−0.086
Δ(Ge(U)-Ge(D))	0.456	0.313	0.312
% Area (Ge(B))	1.3%	28%	38%
**Si 2p**			
Δ(Si(S)’-Si(B))	0.120	0.110	0.132
Δ(Si(S)”-Si(B))	0.056	0.116	0.070
% Area (Si(B))	48%	54%	54%

## Data Availability

Data presented in this article is available on request from the corresponding author.
